# The microRNA *miR-243* directs poly(UG) modification of a somatic mRNA in *C. elegans*

**DOI:** 10.17912/micropub.biology.001754

**Published:** 2025-08-13

**Authors:** David D. Lowe, Martin Newman, Gary Ruvkun, Scott G. Kennedy

**Affiliations:** 1 Genetics, Harvard Medical School, Boston, MA, USA; 2 St. Jude's Research Hospital, Memphis, TN, USA; 3 Molecular Biology, Massachusetts General Hospital, Boston, MA, USA

## Abstract

*C. elegans* RDE-3 adds poly UG tails to mRNAs targeted for silencing by the dsRNA-initiated RNA interference (RNAi) pathway. RDE-3 can also add p(UG) tails to some endogenous cellular mRNAs. Mechanisms directing RDE-3 to pUGylate specific mRNAs are not understood. Here we show that the miRNA *miR-243* directs pUGylation of an intestine-specific mRNA *y47h10a.5* , which leads to silencing of the mRNA. The data show that genome-encoded small regulatory RNAs are one mechanism by which RDE-3 can be directed to pUGylate specific mRNAs for gene regulation.

**Figure 1. RDE-3-dependent silencing of intestinal y47h10a.5 mRNAs:**
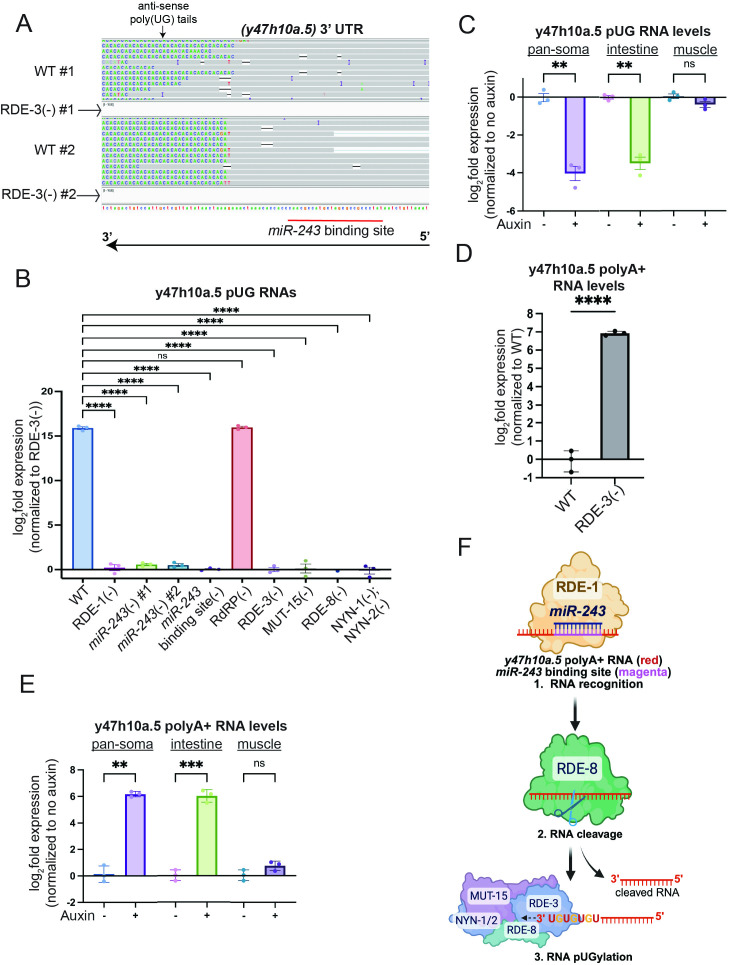
**(A)** IGV screenshots of *y47h10a.5* pUG RNAs sequenced from WT or RDE-3 (-) animals. n=2. Grey indicates sequence matching genome. Non-templated pUG tails are shown as AC repeats, due to gene orientation. *miR-243* binding site is indicated in red. **(B)** pUG RT-PCR to quantify *y47h10a.5* pUG RNA in *rde-1 ( ne300 )* [ RDE-1 (-)], *miR-243 ( n4759 )* [ *miR-243 (-) #1* ], *miR-243 ( gg1162 )* [ *miR-243 (-) #2* ], *y47h10a.5 ( gg1163 )* [ *miR-243 binding site(-)* ], *rrf-1 ( pk1417 ) ego-1 ( gg685 )* [RdRP(-)], *rde-3 ( ne3370 )* [ RDE-3 (-)], *mut-15 ( tm1358 )* [ MUT-15 (-)], *rde-8 ( tm2252 )* [ RDE-8 (-)], and *nyn-1 ( tm5004 ); nyn-2 ( tm4844 )* [ NYN-1 (-); NYN-2 (-)] animals. Data are normalized to *gsa-1* , which harbors a genome-encoded UG repeat. Unpaired, one-way ANOVA. n=3. **(C-E)** qRT-PCR using *y47h10a.5* pUG RNA **(C)** or *y47h10a.5* polyA+ RNA-specific primers **(D-E)** after auxin-mediated tissue-specific depletion of RDE-3 in the entire soma (pan-soma), intestine, or muscle **(C and E)** or in WT or *rde-3 ( ne3370 )* [ RDE-3 (-)] **(D)** animals. **(C)** *y47h10a.5* pUG RNA was normalized to *gsa-1* and **(D-E)** *y47h10a.5* polyA+ RNA was normalized to *eft-2* and *cdc-42* . Paired, two-tailed t-test. n=3. **(F)** Model for *y74h10a.5* RNA silencing by pUGylation. The RDE-1- *miR-243* complex recognizes *miR-243* binding site in the 3' UTR of *y47h10a.5* , recruiting the pUGasome, which directs RDE-8-dependent endonucleolytic cleavage and RDE-3-dependent pUGylation in intestinal cells. ns - not significant,* p<0.05, ** p<0.01, *** p<0.001, **** p<0.0001.

## Description

*C. elegans* employs an endogenous RNA interference (endoRNAi) pathway to regulate gene expression in its germline and soma (Zhang et al., 2011; Phillips et al., 2012). One somatic endoRNAi target is the intestinally expressed transcript *y47h10a.5* , which encodes a protein with homology to RAI1-like 5' mRNA decapping exonucleases (Gu et al., 2009; Corrêa et al., 2010; Chang et al., 2012). Endo RNAi of *y47h10a.5* is initiated by an atypical microRNA *miR-243* , which loads onto the Argonaute protein RDE-1 and binds with perfect complementarity to the 3′ untranslated region (UTR) of *y47h10a.5* ( Figure 1A ) (Gu et al., 2009; Corrêa et al., 2010). This interaction triggers RNA-dependent RNA polymerase (RdRP)-mediated secondary siRNA production, via a mechanism that remains poorly understood, to silence the *y47h10a.5* mRNA *in trans* (Gu et al., 2009; Corrêa et al., 2010).

We asked if the *y47h10a.5* mRNA was pUGylated during normal growth and development. Indeed, nanopore sequencing and qRT-PCR identified RDE-3-dependent pUG tails appended 20–30 nucleotides 3' to the predicted *miR-243* binding site in the 3′ UTR of *y47h10a.5* ( Figure 1A- C). In *rde-3* mutants, or after auxin-based depletion of RDE-3 in intestinal cells, the *y47h10a.5* mRNA became overexpressed ( Fig. 1D /E). Taken together, the data indicate that RDE-3 pUGylates the *y47h10a.5* mRNA in intestinal cells and that this pUGylation drives silencing of *y47h10a.5* .

Animals lacking *miR-243* , the *miR-243* binding Ago RDE-1 , or the *miR-243* binding site in *y47h10a.5* failed to produce *y47h10a.5* pUG RNAs ( Figure 1B ). Thus, *miR-243* directs pUGylation of *y47h10a.5 .* Additionally, current models posit that RDE-8 cleaves mRNAs targeted by RDE-1 and RNAi (Tsai et al., 2015; Shukla et al., 2020) and interacts physically with RDE-3 (Lowe et al., 2025). RDE-8 was also required for *y47h10a.5* pUGylation ( Figure 1B ). Three proteins, NYN-1 , NYN-2 , and MUT-15 are thought to assemble into a larger complex, termed the pUGasome, with RDE-8 and RDE-3 (Lowe et al., 2025). NYN-1 /2 and MUT-15 were also required for *y47h10a.5* pUGylation ( Figure 1B ), suggesting that *y47h10a.5* pUGylation depends upon the pUGasome. RdRP, however, was not required for *y47h10a.5* pUGylation, suggesting that pUGasome activity precedes RdRP-based siRNA production ( Figure 1B ). The results support a model in which *miR-243* – RDE-1 recruits the pUGasome to cleave, pUGylate, and silence the *y47h10a.5* RNA ( Figure 1F ). Thus, pUGylation can be directed by a single trans-acting, genomically encoded small RNA. It will be of interest to assess if other genome-encoded small regulatory RNAs have similar abilities.

## Methods

*Strains*

All *C. elegans* strains were grown at 20 °C, derived from the Bristol N2 strain, and grown on normal growth medium with OP50 bacteria unless otherwise stated. Strains and all the sequences for oligonucleotides are listed in reagents.

*RNA Extraction*

Animals were collected in TRIzol Reagent and freeze-thawed at least three times. RNA was extracted with Isoamyl-alcohol and chloroform twice or with RNA clean and concentrator with on-column DNAse digestion (Zymo Research, R1014). RNA was quantified using a Nanodrop2000 to determine concentration.

*Library Preparation for Nanopore Sequencing*

Streptavidin MyOne T1 Dynabeads and *biotin-(AC)25* oligonucleotides for RNA pulldown were prepared according to manufacturer's instructions except sodium chloride was replaced with lithium chloride for the buffers. RNAseOUT was used as an RNAse inhibitor throughout the protocol. Spike-in RNA ( *gfp(UG)18* ) was synthesized using the Megascript T7 kit (Invitrogen, AM1334) and purified using the RNA Clean Concentrator (Zymo Research, R1014). 60 ug of total RNA and 100 pg of spike-in RNA in non-stick tubes (Invitrogen, AM12400) with RNAseOUT (Invitrogen, 10777019) were prewarmed at 65 °C for 5 mins. 100 ug of biotin-poly(AC)12 beads were added and incubated with constant rotation at 55 °C for 30 mins. The beads were washed with a washing buffer, then eluted with nuclease-free water and heat at 75 °C. The elution was resuspended in a new non-stick tube and pUG RNAs were enriched for a second time and eluted using the RNA clean concentrator (Zymo Research, R1014) for splint ligation. A nanopore compatible DNA donor and splint adapter were annealed on a thermocycler with heating at 95°C -1°C per second. The purified pUG RNA and annealed splint adapter was ligated at 25 °C overnight with T4 DNA Ligase (high concentration; NEB) and 20% PEG8000. After overnight incubation, Lambda Exonuclease (NEB, M0293L) and USER enzyme (NEB, M5505L) were added with Exo I buffer and incubated at 37 °C for 20 minutes. RNA was purified using the RNA clean and concentrator kit (Zymo Research, R1014) for reverse transcription with Maximus H Minus Reverse transcriptase (Thermo Fisher, EP0742) and Strand Switching Primer II or SSP II with UMIs (Oxford Nanopore) to generate cDNA according to cDNA-PCR barcoding protocol (Oxford Nanopore). Reverse transcription was performed at 42 °C for 90 mins and Longamp Taq Polymerase (NEB, M0287L) was used to amplify for 18 cycles before Exo I (NEB, M0293L) treatment and Ampure XP bead (Beckman Coulter, A63881) cleanup (0.65X) for library purification.

*Nanopore Sequencing*

The library was pooled and prepared according to Nanopore protocol. Briefly, the pooled library was loaded into MinION r9.4.1 flow cells on a MinION Mk1C machine (Oxford Nanopore). Sequencing was set to run for at least 24 hours, barcoding was turned on with no trimming, and quality score filter of 9. Guppy high accuracy base calling model was used. FASTQ files that passed the quality score filter were in the fastq_pass folder. The FASTQ files were merged using *cat* *.fastq. Next, pychopper (Oxford Nanopore) was used to identify and orient full-length transcripts. The PCS111_primers.fas was modified as following:

>VNP

TTGCCTGTCGCTCTATCTTC

>SSP

TCTGTTGGTGCTGATATTGCTTT

Pychopper was used with the following settings: -m edlib -p -k PCS111. The output FASTQ files were then merged and filtered for TG repeats using the following:

grep -B1 -A2 --no-group-separator -E .GTGTGTGTGT.{1\}. Then, the filtered FASTQ files were then used to map to the *C. elegans* genome (version WS245) using minimap2 -uf k14 (version 2.22). The resulting SAM files were converted to BAM files that were indexed and sorted using samtools (version 1.9). To analyze visually, mapped reads were loaded onto the integrated genome viewer or IGV (version 2.12.2, Broad institute). Spike-in RNAs served as controls and were detected in all libraries (Lowe et al., 2025).

*qRT-PCR Assay*

Total RNA were reverse transcribed with the Superscript IV kit (Thermo Fisher) according to the manufacturer's protocol. A poly(AC)12 with 5' template sequence encoding an amplification sequence was used to detect pUG RNAs from 5 ug of total RNA. An oligo(dT)20 was used to reverse transcribe polyadenylated mRNAs from 1 ug of total RNA. 1:10 diluted cDNA was mixed with SYBR Green Master Mix (Biorad, 1725121) and primers for qPCR according to manufacturer's recommendation in a CFX Connect machine (Biorad). The CT values were used to calculate relative expression levels using the -2 ^ΔΔ^ method. To quantify *y47h10a.5* RNAs, primers targeting the coding sequence were used. To quantify mRNAs, primers targeting *eft-2* and *cdc-42* were used as housekeeping genes. To quantify pUG RNAs, *gsa-1* primers were used as control.

*Auxin-mediated degradation*

Animals containing degron tagged RDE-3 with TIR1 expression in either the germline or soma were egg prepped onto 1 mM auxin plates (Lowe et al., 2025). Animals were grown for 4 days before collection for RNA extraction and qRT-PCR measuring *y47h10a.5* and control *gsa-1* pUG RNAs as well as *y47h10a.5* , *eft-2* , and *cdc-42* for mRNAs.

*Generative AI and AI-assisted technologies*

During the preparation of this work, the authors used GPT-4o to improve the readability of certain sentences. After using this tool/service, the authors reviewed and edited the content as needed and took full responsibility for the content of the publication.

## Reagents

*C. elegans* strains used in this study.

**Table:** 

**Strain name**	**Genotype**	**Source**
N2	wild-type	CGC
YY1449	*rde-3 ( ne3370 ) I *	CGC
YY1868	*rde-3 ( gg693 ) I; ieSi57 [eft-3p::TIR1::mRuby:: unc-54 3'UTR + Cbr-unc-119 (+)] II *	Kennedy Lab
YY2671	*rde-3 ( gg693 ) I; ieSi57 [myo-2p::TIR1::mRuby:: unc-54 3'UTR + Cbr-unc-119 (+)] II *	Kennedy Lab
YY2672	*rde-3 ( gg693 ) I; ieSi57 [ges-1p::TIR1::mRuby:: unc-54 3'UTR + Cbr-unc-119 (+)] II *	Kennedy Lab
YY1689	*rrf-1 ( pk1417 ) ego-1 ( gg685 ) I *	Kennedy Lab
GR1747	*mut-15 ( tm1358 ) I *	CGC
YY1740	*nyn-1 ( tm5004 ) I; nyn-2 ( tm4844 ) IV *	Kennedy Lab
YY1739	*rde-8 ( tm2252 ) IV *	Kennedy Lab
MT15454	*miR-243 ( n4759 ) IV *	CGC
YY2696	*miR-243 ( gg1162 ) IV *	This work
YY2697	*y47h10a.5 ( gg1163 ) I *	This work
WM45	*rde-1 ( ne300 ) V *	CGC
NL1810	*mut-16 ( pk710 ) I *	CGC

Oligonucleotides used in this study.

**Table:** 

**Name**	**Sequence (5' to 3')**
poly(AC) RT primer	GCTATGGCTGTTCTCATGGCACTTGCCTGTCGCTCTATCTTCACACACACACACACACAC
Nanopore ligation primer	/5Phos/ GAAGATAGAGCGACAGGCAAG /3SpC3/
poly(AC)splint 1	ACTTGCCTGTCGCTCTATCTTC ACACACACACACACACACACA /3ddC/
poly(AC)splint 2	ACTTGCCTGTCGCTCTATCTTC CACACACACACACACACACA /3ddC/
adapter 1 forward	GCTATGGCTGTTCTCATGGC
adapter 2 forward	ACTTGCCTGTCGCTCTATCTTC
*gsa-1* pUG PCR forward 1	GAGTTCTACGATCACATTCT
*gsa-1* qPCR forward	AATATCCACGGCGAACAGCG
*gsa-1* qPCR reverse	GTCGTAGATGAATGCGTTGGATG
*y47h10a.5* pUG PCR forward 1	TCTGTCGCAGAGTTCCGTGA
*y47h10a.5* qPCR forward	GATCGCGGCGGGATATTAGAC
*y47h10a.5* qPCR reverse	CCGTCACTATTTGGCCAGTTAATC
*eft-2* qPCR forward	CTGCCCGTCGTGTGTTCTAC
*eft-2* qPCR reverse	TCCTCGAAAACGTGTCCTCTT
*cdc-42* qPCR forward	TCCACAGACCGACGTGTTTC
*cdc-42* qPCR reverse	AGGCACCCATTTTTCTCGGA
